# 毛细管电泳-无鞘流电喷雾质谱用于药物分析

**DOI:** 10.3724/SP.J.1123.2022.07015

**Published:** 2023-02-08

**Authors:** Hanzhi ZHANG, Feng LI, Jingwu KANG

**Affiliations:** 1.生命有机化学国家重点实验室，中国科学院上海有机化学研究所，上海 200032; 1. State Key Laboratory of Bioorganic and Natural Products Chemistry，Shanghai Institute of Organic Chemistry，Chinese Academy of Sciences，Shanghai 200032，China; 2.西安市食品安全检测与风险评估重点实验室，西安文理学院化学工程学院，陕西 西安 710065; 2. Xi’an Key Laboratory of Food Safety Testing and Risk Assessment，School of Chemical Engineering，Xi’an University，Xi’an 710065，China; 3.弈柯莱生物科技（上海）股份有限公司，上海 200241; 3. Abiochem Biotechnology Co.，Ltd.，Shanghai 200241，China

**Keywords:** 无鞘流接口, 非水毛细管电泳-质谱, 金箔包裹的电喷雾电极, 酪氨酸激酶抑制剂, sheathless interface, nonaqueous capillary electrophoresis-mass spectrometry （NACE-MS）, gold foil-wrapped electrospray ionization emitter, tyrosine kinase inhibitors

## Abstract

毛细管电泳-质谱联用技术具有分离效率高、检测灵敏度高、样品消耗量少，可同时提供样品的结构信息等优点，成为复杂样品分离分析的强有力工具。但是，毛细管电泳与质谱联用的接口技术依然未能很好的解决。为了拓展我们发展的金箔包裹的毛细管电泳分离柱尖端直接作为喷雾电极和无鞘流质谱接口的应用，本文报道了用无鞘流接口毛细管电泳-电喷雾质谱联用（CE-ESI-MS）分析5种酪氨酸激酶抑制剂（舒尼替尼、甲磺酸伊马替尼、吉非替尼、达沙替尼、埃罗替尼）的研究结果。这种接口集分离与电喷雾离子化于一根毛细管中，制作简单，成本低廉，且可批量制作。实验发现采用非水毛细管电泳分离模式不仅可以对5种酪氨酸激酶抑制剂实现基线分离，而且可以获得稳定的质谱信号。考察了电解质溶液组成对分离效果的影响，得到优化的背景电解质组成，即含2%（v/v）乙酸及5 mmol/L乙酸铵的乙腈-甲醇（80∶20， v/v）混合溶剂。在优化的条件下，5种激酶抑制剂可以得到基线分离，无鞘接口也可以长时间保持稳定的电喷雾，分析物的保留时间日内、日间重复性（RSD值）分别小于0.5%和0.8%，接口批次间的RSD值小于2.6%。与水相分离条件下的CE-MS对比，非水相条件下的5种酪氨酸激酶抑制剂的分离柱效更高，检测灵敏度更高，绝对检出限达到amol级。此外，采用无鞘流CE-MS分析了各类有机酸（千层纸素A、丹酚酸C和迷迭香酸）和脂溶性的大环内酯类抗生素（阿奇霉素、红霉素和环孢素A），均可以获得良好的分离效果和质谱检测结果。

## Abstract (en)

Capillary electrophoresis-mass spectrometry （CE-MS） combines the advantages of capillary electrophoresis， such as the high separation efficiency and low sample consumption， and the high detection sensitivity of mass spectrometry and the ability for providing the structural information for structure elucidation of unknown components. However， the interface technology for coupling capillary electrophoresis and mass spectrometry is still not well resolved. In the present work， we explored the application of the sheathless CE-MS interface which was prepared by gold foil-wrapped CE separation column tip directly as a spray electrode for the analysis of five tyrosine kinase inhibitors， namely sunitinib， imatinib mesylate， gefitinib， dasatinib and erlotinib. This interface integrates separation and electrospray ionization in one capillary， which is easy to manufacture， low in cost， and can be produced in batches. We found that using the nonaqueous CE separation mode can not only achieve baseline separation of five tyrosine kinase inhibitors， but also obtain stable mass spectrometry signals. First， we investigated the effect of the electrolyte solution composition on the separation. The optimized background electrolyte composition was obtained： 2% （v/v） acetic acid and 5 mmol/L ammonium acetate in acetonitrile-methanol （80∶20， v/v）. Under optimized conditions， the five kinase inhibitors could be baseline separated， meantime， the sheathless interface could also maintain stable electrospray for a long time. The relative standard deviation （RSD） values of the intraday and interday repeatability of the analyte retention times were less than 0.5% and 0.8%， respectively， and the RSD value between interface batches is less than 2.6%. Compared with CE-MS with aqueous phase， the separation column efficiency of the five tyrosine kinase inhibitors under nonaqueous phase conditions is higher， the detection sensitivity is higher， and the absolute detection limit reaches amol level. In addition， we evaluated the sheathless interface with various organic acids， such as palaflin A， salvianolic acid C， and rosmarinic acid， as well as hydrophobic macrolide antibiotics， azithromycin， erythromycin， and sporin A， good separation effect and mass spectrometric detection results can be obtained.

作为一种高效的液相分离技术，毛细管电泳（CE）具有分离时间短、分离柱效高和样品用量少等优点^[1⇓-3]^。毛细管电泳-质谱联用技术（CE-MS）具有分离效率高、检测灵敏度高、样品消耗量少，并且可同时提供样品的结构信息等优点，成为复杂样品分离分析的强有力工具^[4⇓-6]^。非水毛细管电泳（NACE）是毛细管电泳分离模式的一种延伸，能够分离水溶性差或在水相介质中分离效果较差的分析物^[7,8]^。同时由于NACE一般采用高挥发性、表面张力小的有机溶剂，电泳过程产生较小的电流和焦耳热，也非常适合与高灵敏度的MS联用。近年来，非水毛细管电泳-质谱（NACE-MS）在复杂生物样品、环境样品、食品、手性药物和农药制剂的分析中也受到广泛关注^[9,10]^。与水相CE-MS相比，NACE-MS的背景电解质（BGE）中大量的有机溶剂提高了电喷雾离子源（ESI）效率，能够稳定喷雾电流，降低背景噪声的信号，提高检测灵敏度。同时NACE-MS可以兼容多种有机溶剂、手性选择剂或表面活性剂等，提高了CE的分离选择性，扩大了CE-MS的应用范围^[11,12]^。Chen等^[9]^也利用实验室自己发展的CE-MS接口技术，开发了一种定量NACE-MS/MS分析方法，应用于大黄中3种活性成分大黄素、大黄酚和芦荟大黄的分析。Britz-McKibbin等^[10]^在利用NACE-MS技术进行血液中脂类代谢组学研究中，基于精确相对分子质量和相对迁移时间实现了279种血脂特征成分分析，结果证明该技术极大地扩展了传统水相CE-MS用于代谢组学研究的覆盖范围，可以构建大规模脂质组学研究的高效平台。

近年来，CE和MS联用系统的研究层出不穷，而接口技术的研制至关重要，目前主要的接口技术包括鞘流、液联接和无鞘流接口等^[13]^。其中鞘流接口的灵活性使其被更广泛的应用，但鞘流组成的多变性以及对样品的稀释也限制了该技术的发展^[14]^。而无鞘流接口可更有效地提高分析灵敏度，最大限度地减少样品稀释。最近，我们课题组报道了一种无鞘流接口技术，在分离毛细管末端通过氢氟酸蚀刻成对称性锥形尖端，再包裹导电涂层金箔即制备成电喷雾电极。其中分离与电喷雾离子化在一根毛细管上实现，该无鞘流CE-MS系统已成功应用于生物碱类、小分子阴离子以及（糖）蛋白酶解产物的分离分析中^[15]^。

本文利用实验室自己发展的无鞘流接口技术，建立了同时分析5种酪氨酸激酶抑制剂（结构式见图1）的NACE-MS分析方法，用于拓展我们发展的无鞘流CE-MS联用技术的应用。首先利用NACE-UV优化了毛细管电泳的分离条件，包括背景电解质中的乙酸含量、盐浓度及有机溶剂组成等。与水相CE-ESI-MS相比，NACE-MS分析时间更短、检测灵敏度更高。同时NACE-MS系统的迁移时间在日内、日间及接口批次之间的重复性良好，对上述物质的绝对检出限达到amol级别。此外，进一步将NACE-MS扩展至有机酸类化合物和大环内酯类抗生素的分离分析中。

**图1:**
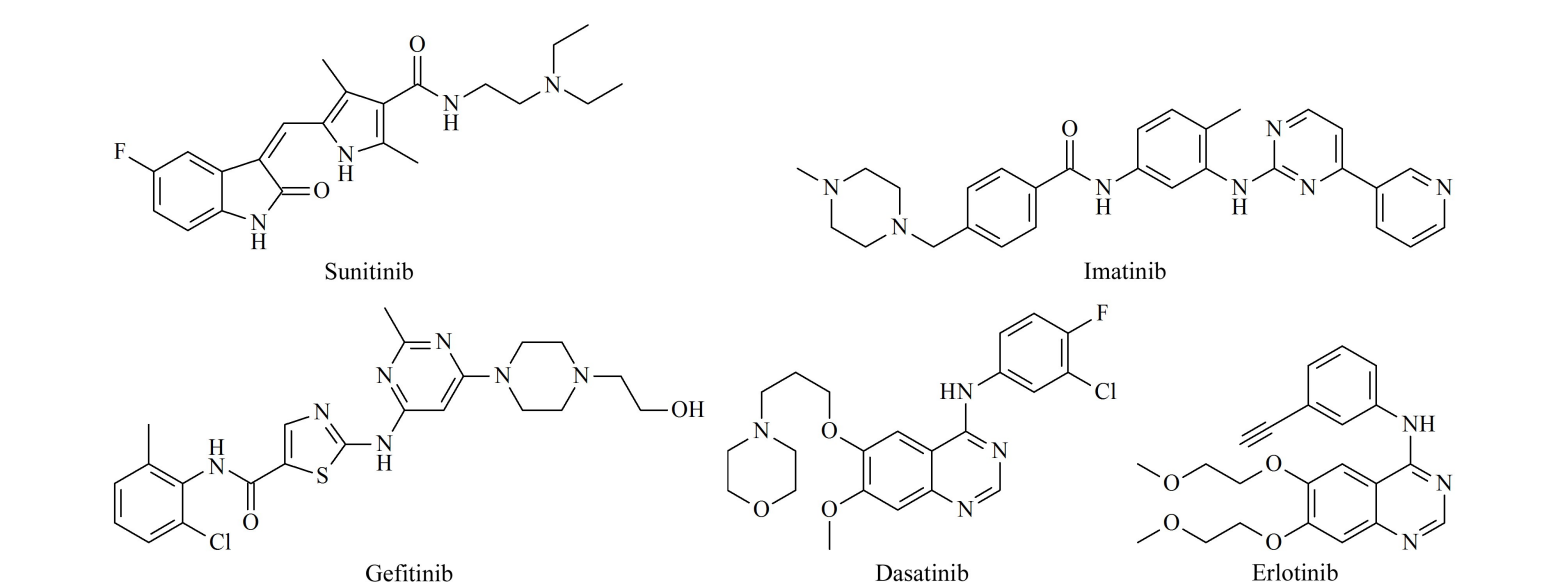
5种酪氨酸激酶抑制剂的化学结构式

## 1 实验部分

### 1.1 仪器、试剂与材料

毛细管电泳仪（Agilent公司），配置DAD检测器； LCQ Fleet ion-trap质谱仪（美国ThermoFisher Scientific公司）。CE与质谱仪经实验室研发的无鞘流接口实现联用，质谱图通过配有Xcalibur系统的计算机采集，电喷雾电极距质谱的位置可以通过*x-y-z*三维架调整。

舒尼替尼（sunitinib）、伊马替尼（imatinib）、吉非替尼（gefitinib）、达沙替尼（dasatinib）、埃罗替尼（erlotinib）（南京安格医药化工有限公司）；千层纸素A（oroxylin A）、丹酚酸C（salvianolic acid C）、迷迭香酸（rosmarinic acid）（上海源叶生物科技有限公司）；阿奇霉素（azithromycin）、红霉素（erythromycin）（上海TCI公司）；环孢素A（cyclosporin A）（浙江八达通生物化工）。甲醇、乙腈（德国Merck公司）；乙酸、甲酸铵、乙酸铵、聚-*N*，*N*'-二甲基溴化己铵（海美溴铵，hexadimethrine bromide， HDB）、磺化葡聚糖（dextran sulfate， DS， *M*_r_ 500000）（美国Sigma-Aldrich公司）；氢氟酸（HF）、二甲基亚砜（DMSO）（上海凌峰化学试剂有限公司）。实验用水为超纯水（18.2 MΩ·cm），由Milli-Q超纯水系统制备（美国Millipore公司）。CE实验中所需溶液使用前均由0.22 μm滤膜过滤。

### 1.2 样品溶液配制

NACE-MS样品溶液：准确称取舒尼替尼、伊马替尼、吉非替尼、达沙替尼、埃罗替尼溶解于含有5 mmol/L乙酸铵的乙腈-甲醇（80∶20， v/v）混合溶剂中，配制储备液质量浓度为0.5 mg/mL，混合后稀释至0.1 mg/mL备用；准确称取千层纸素A、丹酚酸C、迷迭香酸，溶于含有5 mmol/L乙酸铵的乙腈-甲醇（80∶20， v/v）混合溶剂中，混合后稀释至0.2 mg/mL备用；准确称取阿奇霉素、红霉素、环孢素A溶于含有5 mmol/L乙酸铵的乙腈-甲醇（80∶20， v/v）混合溶剂中，混合后稀释至50 μg/mL备用。

CE-MS样品溶液：准确称取舒尼替尼、伊马替尼、吉非替尼、达沙替尼、埃罗替尼溶解于含20 mmol/L甲酸铵的50%（v/v）乙腈水溶液中，配制储备液的质量浓度为0.5 mg/mL，混合后稀释至0.1 mg/mL备用。

### 1.3 实验条件

#### 1.3.1 无鞘流接口的加工

自制的NACE-ESI-MS无鞘流接口的结构如图2a所示。无鞘流接口包括分离毛细管和金箔包裹的电喷雾喷针，分离毛细管末端经氢氟酸刻蚀后形成对称性尖端，包裹金箔后可以使毛细管尖端导电，成为喷雾电极，样品在电场中离子化后进入质谱检测。该接口简单且易制作，与鞘流接口相比，避免了鞘流液的稀释，提高了检测灵敏度。

**图2:**
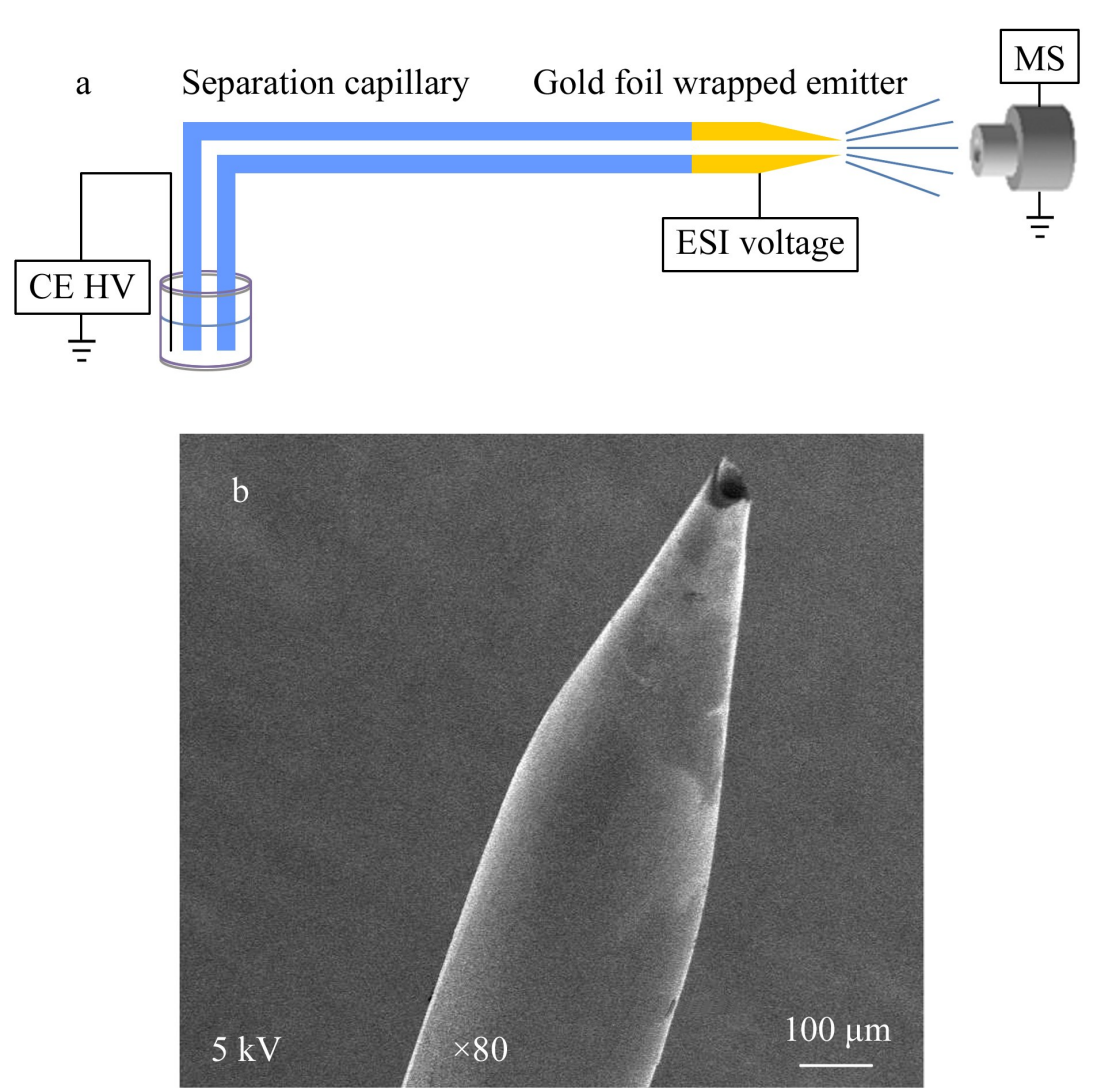
（a）无鞘流NACE-ESI-MS系统示意图及（b）氢氟酸刻蚀方法得到的对称型石英毛细管喷针的SEM图

无鞘流接口集分离与电喷雾电极于一体，关键在于制作金箔包裹的电喷雾喷针，该接口的制作过程包括如下两个步骤^[15]^：（1）改良石英毛细管喷针制备方法：采取一种氢氟酸刻蚀熔融石英毛细管成喷针的方法，烧去毛细管末端5 mm聚酰亚胺涂层，浸到40%氢氟酸液面下约2 mm，内部通氮气，防止氢氟酸进入管内；通过扫描电镜记录刻蚀不同时间后毛细管尖端的状况；（2）制备金箔包裹的电喷雾质谱电极：将金箔（9.2 cm×9.2 cm）裁剪成大小合适的条状（1.2 cm×1.5 mm），通过环氧胶裹覆在毛细管喷针上。制作成本及时间大幅度降低，每张金箔可用于制作近400根喷针，每根包裹时间在3 min以内。

#### 1.3.2 CE-MS条件

弹性石英毛细管柱：70 cm×50 μm I. D.， 360 μm O. D. （Polymicro Technologies， USA）；分离电压：12 kV；压力进样：2.5 kPa×2.5 s；电喷雾电压2.2 kV。NACE-MS缓冲液：含2%乙酸和5 mmol/L乙酸铵的乙腈-甲醇（80∶20， v/v）混合溶剂；CE-MS缓冲液：含20 mmol/L甲酸铵的50%（v/v）乙腈水溶液（pH 3.0）。

## 2 结果与讨论

### 2.1 无鞘流接口的制作

制作接口时，氢氟酸会浸润毛细管外管壁，在液面以上的毛细管表面形成弯月形刻蚀层，该刻蚀层厚度从下到上逐渐变薄，即在包有聚酰亚胺涂层的毛细管处最薄。液面以下的毛细管被刻蚀掉后，会逐渐依据刻蚀层厚度刻蚀液面以上的毛细管。由于刻蚀速度不同，当液面以上的毛细管消失后，会在包有聚酰亚胺涂层的毛细管处自动得到成对称型的尖端，如图2b所示，壁厚减小，内径并没有变化，外径缩小至约5 μm，电泳时从毛细管流出的液体可以及时接触到导电涂层产生电喷雾。刻蚀时间会因去掉聚酰亚胺涂层的毛细管长度而有所变化，一般在120~180 min。由于对称型尖端是自动刻蚀得到，制备的电喷雾喷针具有很高的重复性，可批量制作；喷针内径不受影响，不会干扰分离行为。

### 2.2 NACE-MS条件的优化

本文选择5种酪氨酸激酶抑制剂的混合物作为测试样品，采用HDB-DS对毛细管内壁进行动态双涂层修饰，并考察了BGE溶剂组成、酸及盐的含量等因素对NACE分离的影响。

#### 2.2.1 BGE条件的选择

在NACE中，电泳缓冲液中有机溶剂的种类和含量对动态涂层的稳定性及电渗流的调控具有明显影响。对于有机溶剂的选择，需要考察黏度（*η*）、介电常数（*ε*）、表面张力系数（*γ*）、质子自递常数（p*K*_auto_）、极性及沸点等参数的影响。其中，黏度和介电常数是影响电泳淌度（*μ*_eq_）和电渗流（*μ*_eo_）的重要因素，如公式（1）、（2）所示。如果改变了溶剂种类，分析物离子的溶剂化半径、*ε*和*η*也随之改变。同时*μ*_eq_和*μ*_eo_会随着*ε*/*η*比值的增大而增大，为提高分析速度应选择*ε*/*η*比值较大的有机溶剂。对比分析各类常见溶剂的*η*和*ε*值，乙腈是非常适合作为NACE的良好溶剂。


（1） $\mu_{\mathrm{eq}}=\frac{q}{6 \pi \eta r}=\frac{2 \varepsilon \zeta_{\mathrm{ion}}}{3 \eta}$

（2） $\mu_{\mathrm{eo}}=\frac{\varepsilon \zeta_{\text {wall }}}{\eta}$

其中，*q*，分析物电荷；*r*，分析物的Stokes半径；*ε*，溶剂的介电常数；*ζ*_ion_，分析物离子的zeta电势；*ζ*_wall_毛细管壁的zeta电势；*η*，溶剂的黏度。

实验中，首先尝试采用含有2%（v/v）乙酸和5 mmol/L乙酸铵的乙腈溶液作为BGE分离5种酪氨酸激酶抑制剂，见图3a，伊马替尼和吉非替尼无法实现基线分离。随着甲醇含量的增加，如加入20%甲醇时，即BGE为含有2%（v/v）乙酸和5 mmol/L乙酸铵的乙腈-甲醇（80∶20， v/v）混合溶剂时，各分析物的分离效果得到改善，进一步增加至40%甲醇，对吉非替尼与达沙替尼的分离度没有明显改善。甲醇作为一种质子化极性溶剂，加入到背景电解质中，改变了分析物离子的溶剂化状态，增加了离子与溶剂的氢键作用，提高了分离选择性。然而甲醇的*ε/η*值较乙腈小，CE分离的电渗流减小，分析时间延长。综合考虑，选择加入20%甲醇。

**图3:**
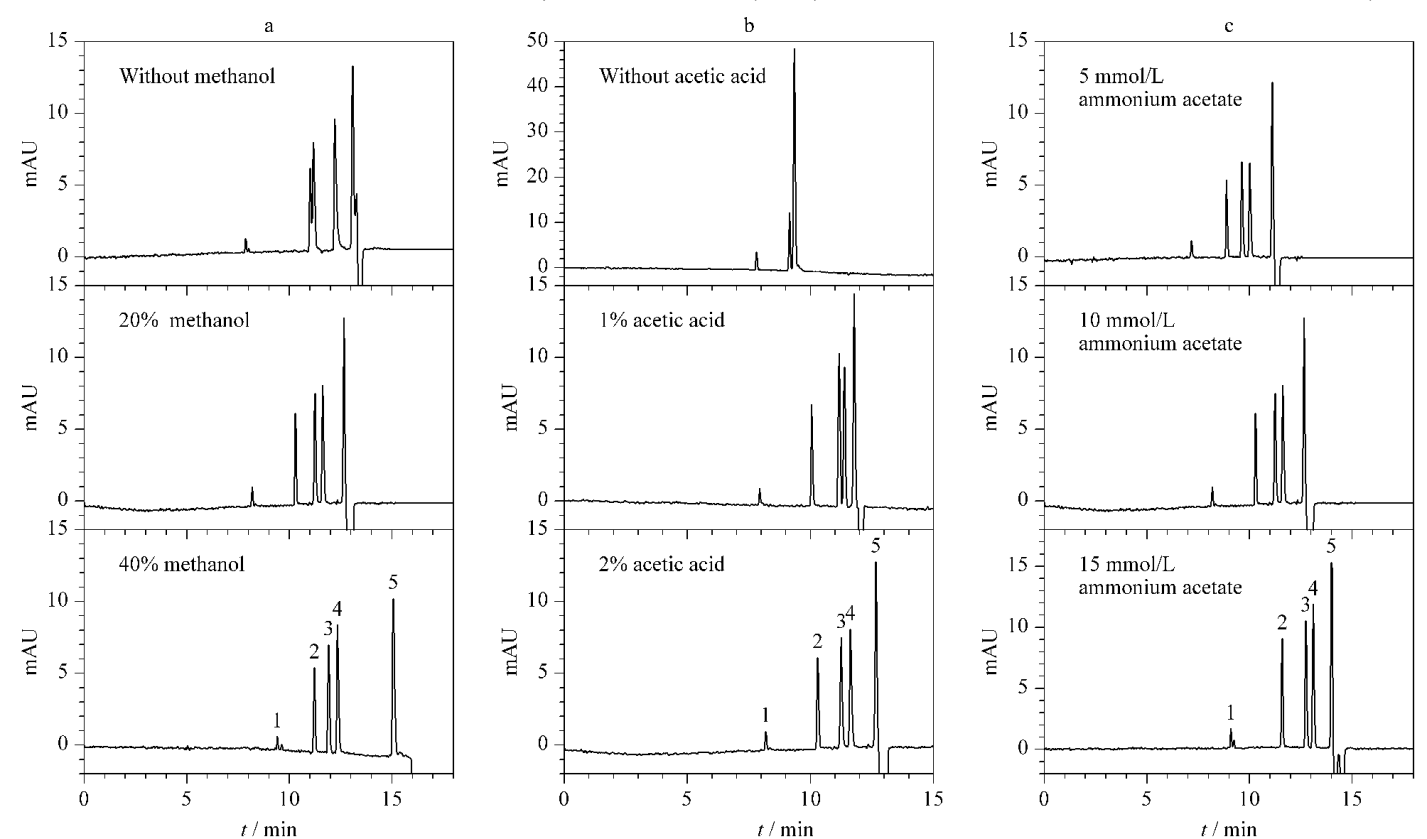
（a）甲醇、（b）乙酸及（c）乙酸铵含量对NACE分离5种酪氨酸激酶抑制剂的影响

不同乙酸含量对5种酪氨酸激酶抑制剂分离的影响如图3b所示。实验表明，当含5 mmol/L乙酸铵的乙腈-甲醇（80∶20， v/v）溶液中不加入乙酸时，5种抑制剂基本无分离。各分析物的分离度随着乙酸含量的增加而逐渐变大，加入2%的乙酸，相较于加入1%乙酸的分离情况，吉非替尼和达沙替尼实现了基线分离。由于所选择的抑制剂含有较多的氮原子，乙酸的加入使待测物的质子化程度增加，大大提高了分离度。但乙酸的加入同时抑制了*μ*_eq_和*μ*_eo_，使得出峰时间略有增加。在保证分离度的基础上，为缩短分析时间，本实验选择在缓冲溶液中加入2%乙酸。

实验进一步考察了不同浓度的乙酸铵溶液对5种激酶抑制剂分离的影响。在背景缓冲液即含2%（v/v）乙酸和不同浓度的乙酸铵的乙腈-甲醇（80∶20， v/v）混合溶剂中增加乙酸铵的浓度（5、10和15 mmol/L），缓冲液中N $H_{4}^{+}$ 和Ac^2-^离子浓度随之增大（见图3c），待测物与电解质之间的库仑作用增强。同时盐浓度的增加改变了毛细管壁的zeta电势，使得*μ*_eo_显著降低，增加了分析时间。在乙酸含量固定的情况下，改变乙酸铵的浓度，吉非替尼与达沙替尼的分离度没有产生明显变化。这说明抑制剂结合酸的能力在该条件下得到饱和，分析物离子之间的相互作用在分离中影响较小。

根据CE-UV对5种抑制剂的分离情况，为避免盐浓度过大造成离子抑制，我们确定了用于NACE-MS联用时的背景电解质溶液组成，选择乙酸铵浓度为5 mmol/L，为达到基线分离，采用乙腈-甲醇（80∶20， v/v）混合有机溶剂，其中含有2%的乙酸。为尽量减少因有机溶剂挥发而造成背景电解质的变化并提高系统的稳定性，应根据峰形及时更换缓冲液。

#### 2.2.2 NACE-MS与水相CE-MS分离情况的比较

我们对比了NACE-MS与水相CE-MS对5种激酶抑制剂的分离效果，其基峰色谱图如图4所示。实验中，以电中性化合物DMSO测试了非水相和水相的电渗流大小，其中NACE的电渗流为170 nL/min，而CE的电渗流为80 nL/min。这表明非水相中的迁移速度大于水相中的迁移速率。这是因为乙腈黏度较低，具有高的*ε/η*值，因此具有较高的电泳淌度，从而可以缩短分析时间。同时实验发现，在两种不同的介质中，5种抑制剂的出峰顺序也发生了变化，这可能是因为分析物与缓冲溶液存在不同的相互作用，包括氢键、离子对及（缔合）偶极-偶极相互作用等。

**图4:**
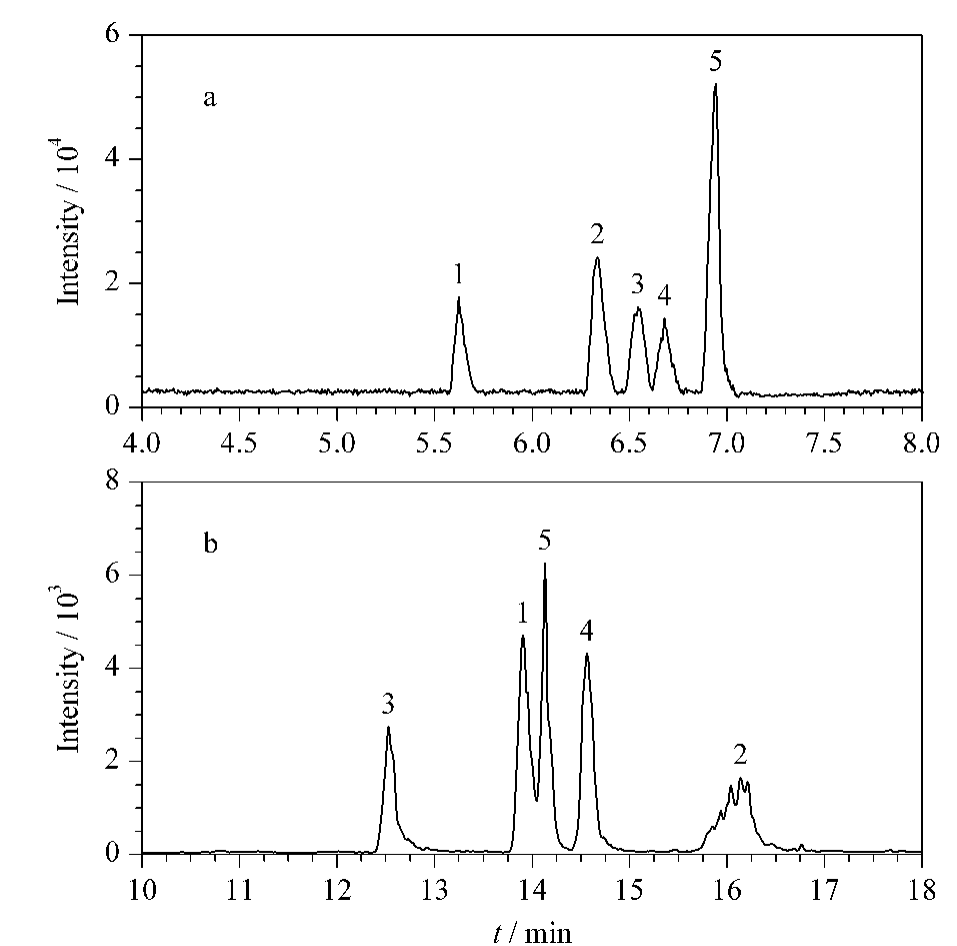
（a）NACE-ESI-MS与（b）水相CE-ESI-MS分离5种激酶抑制剂的电泳图

与水相CE相比，NACE中有机溶剂改善了色谱峰形，如化合物伊马替尼在水相CE-ESI-MS中的峰形较差，这可能是由于较长的分析时间造成后期喷雾不稳定。相同进样条件下，NACE-MS的信号强度是水相CE-MS的10倍，说明NACE的方法具有更高的检测灵敏度。分析原因可能是：一方面NACE中采用较低的盐浓度，降低了高浓度离子对样品信号的抑制作用；另一方面，乙腈和甲醇较低的表面张力使得产生的喷雾更稳定，且样品去溶剂化过程更快，形成更小的带电液滴，从而提高了质谱检测灵敏度。

### 2.3 NACE-MS的方法学评价

以上述5种抑制剂的测试为例，考察了该无鞘流接口的稳定性和重复性，如表1所示。结果表明，其日内、日间RSD值分别小于0.5%和0.8%，与水相CE-ESI-MS的0.8%和2.7%相比，该接口在非水相的分析模式下具有更加稳定的性能。同时对实验室自制的3根无鞘流接口的柱间重复性也进行了测试，RSD均小于2.6%，得到了令人满意的结果。柱间的差异性较小，说明该无鞘流接口有希望作为一种商品化的接口技术用于更广泛的CE-ESI-MS联用系统。

**表1:** 5种酪氨酸激酶抑制剂的检出限及迁移时间的重复性

Compound	LOD/ amol	RSDs of migration time		
		Intraday （*n*=3）	Interday （*n*=3）	Batch-to-batch （*n*=3）
Sunitinib	370	0.48	0.75	2.5
Imatinib	298	0.42	0.27	1.5
Gefitinib	330	0.21	0.32	1.3
Dasatinib	302	0.34	0.51	1.3
Erlotinib	374	0.50	0.74	1.7

Experimental conditions are the same as those in Fig. 4；injection，0.25 kPa×2.5 s.

### 2.4 NACE-MS对其他有机离子化合物的分析考察

生物体内，有很多阴离子化合物如部分药物及其代谢物、有机酸及酸性糖链等。这些物质在体内代谢调节中发挥了很关键的作用。目前，无鞘流CE-MS在该方面的报道极少，这是因为阴离子化合物一般在质谱负离子模式下分析，而喷雾的稳定性较差，给分离鉴定增加了难度。在本工作中，基于实验室自主开发的无鞘流接口，探索一种稳定的背景电解质体系，进一步将NACE-ESI-MS用于阴离子化合物的分析中。我们选择了3种酸性化合物千层纸素A、丹酚酸C和迷迭香酸为检测对象，以含5 mmol/L乙酸铵的乙腈-甲醇（80∶20， v/v）为背景电解质，NACE-ESI-MS电泳图如图5所示。在质谱负离子模式检测，其[M-H]^-^的质谱图如图5所示。实验结果表明，NACE-ESI-MS用于酸性化合物的分离基线平稳，说明质谱负电压的检测模式下，该无鞘流接口可以得到稳定的电喷雾，证明我们开发的NACE-ESI-MS系统适用于阴离子型化合物的检测。

**图5:**
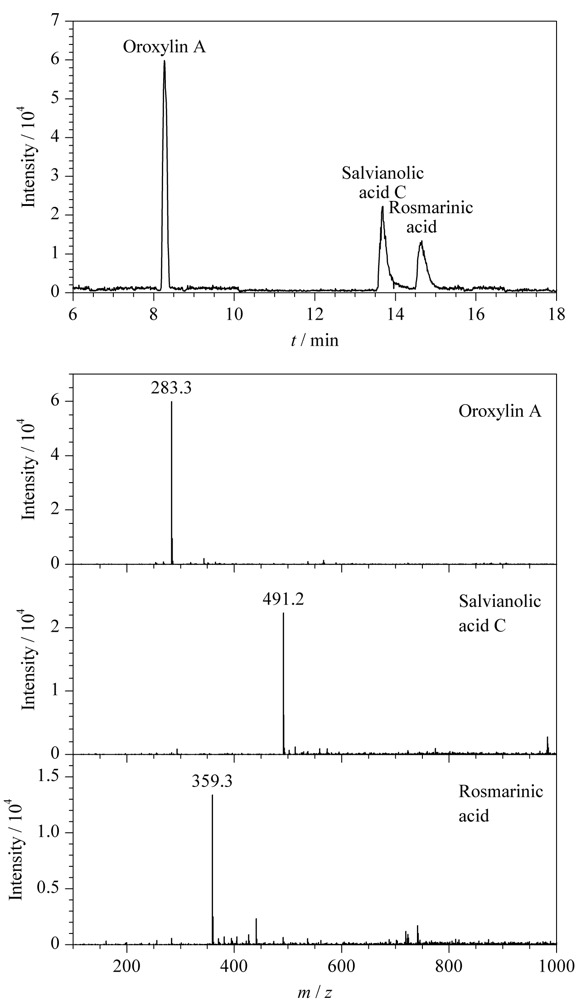
NACE-ESI-MS分离酸类化合物的电泳图及质谱图

进一步将NACE-MS的应用范围扩展至一些难溶性化合物的分析中，选取了3种大环内酯类抗生素化合物阿奇霉素、红霉素和环孢素A，将其溶于乙腈-甲醇（80∶20， v/v）混合溶剂中，NACE-ESI-MS的相关谱图见图6，阿奇霉素和红霉素的基峰是[M+H]^+^的分子离子峰，而环孢素A的基峰为[M+NH_4_]^+^，可能是电喷雾过程中环孢素A结合铵根离子的能力强于氢离子。

**图6:**
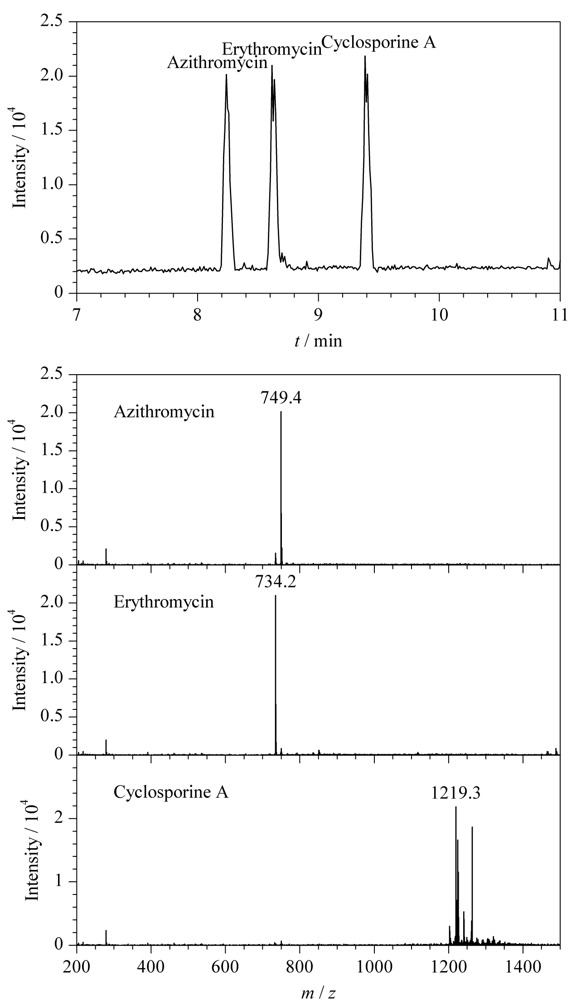
NACE-ESI-MS分离抗生素类化合物的电泳图和质谱图

## 3 结论

本文成功实现了无鞘流NACE-ESI-MS联用系统对于5种酪氨酸激酶抑制剂（舒尼替尼、伊马替尼、吉非替尼、达沙替尼、埃罗替尼）的分析。与水相CE-ESI-MS相比，两者的分离选择性不同，但前者具有一定的优势。结果表明，NACE-ESI-MS特别适合于分离水溶性差而易溶于有机溶剂的物质，同时具有更快的分析速度、更高的分离效率和更加稳定的性能。随着进一步深入的研究，NACE-MS的优异性能会更多地显现出来，它将成为水相CE-MS和HPLC-MS等技术的一种重要补充手段，在更多的领域中发挥重要的作用。

## References

[1] Krait S, Konjaria M L, Scriba G K E. Electrophoresis, 2021, 42(17): 1709 33433919 10.1002/elps.202000359

[2] Voeten R L C, Ventouri I K, Haselberg R, et al. Anal Chem, 2018, 90(3): 1464 29298038 10.1021/acs.analchem.8b00015 PMC5994730

[3] Liu M X, Li X J, Bai Y, et al. Chinese Journal of Chromatography, 2020, 38(3): 317 34213211 10.3724/SP.J.1123.2019.10019

[4] Drouin N, Mever M V, Zhang W, et al. Anal Chem, 2020, 92(20): 14103 32961048 10.1021/acs.analchem.0c03129 PMC7581015

[5] Stolz A, Jooβ K, Höcker O, et al. Electrophoresis, 2019, 40(1): 79 30260009 10.1002/elps.201800331

[6] Wang F, Wang S, Cong H L, et al. Chinese Journal of Chromatography, 2020, 38(9): 1013 34213267 10.3724/SP.J.1123.2020.02025

[7] Walbroehl Y, Jorgenson J W J. Chromatogr A, 1984, 315: 135

[8] Ou W L, Li Y J, Shi D D, et al. Chinese Journal of Chromatography, 2015, 33(2): 152 25989687 10.3724/sp.j.1123.2014.11006

[9] Cheng J H, Wang L Y, Liu W F, et al. Anal Chem, 2017, 89(3): 1411 28208307 10.1021/acs.analchem.6b04944

[10] Azab S, Ly R, Britz-McKibbin P. Anal Chem, 2019, 91(3): 2329 30570251 10.1021/acs.analchem.8b05054

[11] Scriba G K E. J Chromatogr A, 2007, 1159(1): 28 17316665 10.1016/j.chroma.2007.02.005

[12] Schmitt-Kopplin P. Capillary Electrophoresis:Methods and Protocols. Totowa, NJ, USA: Humana Press, 2008

[13] Bonvin G, Schappler J, Rudaz S. J Chromatogr A, 2012, 1267(1): 17 22846629 10.1016/j.chroma.2012.07.019

[14] Zhu L, Zhang N Q, Fang P. Chinese Journal of Analytical Chemistry, 2021, 49(1): 34

[15] Zhang H Z, Lou C L, Li J, et al. J Chromatogr A, 2020, 1624(1): 461215 32540065 10.1016/j.chroma.2020.461215

